# Anxiety and depression in Klinefelter syndrome: The impact of personality and social engagement

**DOI:** 10.1371/journal.pone.0206932

**Published:** 2018-11-09

**Authors:** Anne Skakkebæk, Philip J. Moore, Anders Degn Pedersen, Anders Bojesen, Maria Krarup Kristensen, Jens Fedder, Jens Michael Hertz, John R. Østergaard, Mikkel Wallentin, Claus Højbjerg Gravholt

**Affiliations:** 1 Department of Endocrinology and Internal Medicine (MEA), Aarhus University Hospital, Aarhus, Denmark; 2 Department of Clinical Genetics, Aarhus University Hospital, Aarhus, Denmark; 3 Department of Psychology, The George Washington University, Washington DC, United States of America; 4 Department of Psychology and Behavioral Sciences, Aarhus University, Aarhus, Denmark; 5 Department of Clinical Genetics, Sygehus Lillebaelt, Vejle, Denmark; 6 Department of Mental Health, Odense University Clinic, Odense, Denmark; 7 Centre of Andrology and Fertility Clinic, Department D, Odense University Hospital, Odense, Denmark; 8 Department of Clinical Genetics, Odense University Hospital, Odense, Denmark; 9 Centre for Rare Diseases, Department of Child and Adolescent Medicine, Aarhus University Hospital, Aarhus, Denmark; 10 Department of Linguistics, Cognitive Science and Semiotics, Aarhus University, Aarhus, Denmark; 11 Center of Functionally Integrative Neuroscience, Aarhus University Hospital, Aarhus, Denmark; 12 Department of Molecular Medicine, Aarhus University Hospital, Aarhus, Denmark; Rush University Medical Center, UNITED STATES

## Abstract

Klinefelter syndrome (KS) (47, XXY) is the most common sex chromosome disorder, with a prevalence of 1 in every 660 newborn males. Despite the profound adverse effects of anxiety and depression, and their greater prevalence in KS populations, no research has been conducted to date to identify the determinants of anxiety and depression among patients with KS. We examined the relationships between personality traits, social engagement, and anxiety and depression symptoms among KS patients (n = 69) and a group of male controls (n = 69) matched for age and years of education. KS patients experienced more anxiety and depression symptoms than control participants. Neuroticism was the strongest and most consistent mediator between KS and both anxiety and depression symptoms. This research suggests that neuroticism may play a central role in attention switching, anxiety and depression among patients with Klinefelter syndrome. The central role of neuroticism suggests that it may be used to help identify and treat KS patients at particularly high-risk for attention-switching deficits, anxiety and depression.

## Introduction

Klinefelter syndrome (KS) (47, XXY) is the most common sex chromosome disorder, with a prevalence of 1 in every 660 newborn males [1;2]. KS is associated with a wide spectrum of comorbidities, including an increased risk of a broad range of mental disorders [3–5]. KS patients are more than three times more likely than the general population to be hospitalized with a psychiatric disorder, with a hazard ratio of 3.65 [6]. Two of the most prevalent and debilitating affective symptoms among KS patients are anxiety and depression. Approximately 18% of KS [4] suffer from generalized anxiety. In addition, rates of clinical depression among KS range from 19 to 24% [3;4], with more than two-thirds (68%) of KS patients reporting depressive symptoms[7]. However, despite the profound adverse effects of anxiety and depression, as well as their greater prevalence in KS populations, no research has been conducted to date to identify the predictors of anxiety and depression among patients with KS. Learning about these associations is important for a better understanding of KS patients’ mental health, as well as for improving the effectiveness of interventions to reduce anxiety and depression among those with KS [8].

Personality traits represent an important group of potential predictors of KS patients’ anxiety and/or depression, as they develop early in life, they are stable over time, and they have been linked to various forms of psychopathology [9–14]. Increased neuroticism in the general population is associated with generalized anxiety disorder (GAD) and major depressive disorder (MDD), while conscientiousness and extraversion are both negatively correlated with both GAD and MDD (although the link between extraversion and MDD is less consistent) [13]. In other studies, personality traits have been used to identify individuals at risk for onset of mental illness [15–17], supporting the *vulnerability model* of psychopathology, which hypothesizes that personality traits contribute significantly to the development of psychiatric disorders[18;19]. Previously, we showed that KS is associated with higher levels of neuroticism and lower levels of extraversion, conscientiousness, and openness to experience [20]. However, the question of whether these KS personality profiles make KS patients more vulnerable to psychopathology–particularly anxiety and depression–has yet to be addressed.

In addition to personality traits, social engagement has also been proposed as an important factor in relation to psychopathology [21–24]. Social engagement reflects an individual’s general social skills and experiences, including interpersonal attention and communication. The relationship between social skills and interpersonal relations have been well-documented [25;26], as has the link between interpersonal relations and psychopathology [27–30]. Moreover, positive relations with others have been found to mediate the association between social skills and psychopathology [22]. Deficits in social engagement is characteristic of patients with KS, including deficits in attention switching, imagination, communication and general social skills[20;31], which may help explain KS patients’ increased risk of mental illness in general, and anxiety and depression in particular.

To address these issues, we examined the relationships between personality traits, social engagement, and anxiety & depression symptoms among KS patients and a group of male controls matched for age and years of education. Both zero-order and path analyses were performed to identify direct and indirect relationships between these study variables.

## Materials and methods

### Participants

Patients with verified KS were recruited from endocrinology, genetics and fertility clinics in Denmark. Participants included 69 KS patients with 47,XXY karyotype, with a mean age of 36.4 years (SD = 10.0), and an average of 12.6 years (SD = 2.35) of formal education. At the time of participation, 48 (70%) of the patients with KS were receiving testosterone, and 21 (30%) were not. Fourteen of these 21 KS patients had never received testosterone treatment, while 6 had been treated with testosterone in the past (ranging from 6 month-7.3 years, with an average of 32.5 months). One patient with KS reported being treated with testosterone for an unspecified period in the past.

In addition, 69 matched male controls—with a mean age of 36.4 years (SD = 9.6) and an average of 12.8 years (SD = 2.21) of formal education—were recruited through advertisement in local hospitals, newspapers, fire departments, and other civil service offices. There were no significant differences between KS and control participants in either age or education level (ps >.84). Inclusion criteria required that participants be between the ages of 18 and 60, and exclusion criteria included a history of substance use, and neurological disease or head injury more severe than simple concussion. All participants received oral and written information about the requirements of the study before written consent was obtained, and informed consent was obtained before participation was initiated. The study was approved by The Danish Data Protection Agency and local ethics committee (Region Midtjylland, Denmark number M-20080238) and registered at ClinicalTrials.gov (Clinical trial NCT00999310). Certain neuropsychological data from this research has been presented previously to address separate research questions [20;32;33].

### Measures

#### Personality

Personality traits were assessed using the Revised NEO Personality Inventory (NEO PI-R) short form[34]. This form includes measures of neuroticism (12 items), extraversion (12 items), agreeableness (12 items), conscientiousness (12 items), and openness to experience (12 items). For all items, participants indicated how well each statement described them on a scale form 1 (“very inaccurate”) to 5 (“very accurate”). Items responses within each personality measure were combined to create five personality scores for each participant. Cronbach´s alphas for these personality traits range from 0.70–0.90.

#### Social engagement

Social engagement was assessed using the Autism Spectrum Quotient (AQ) [35]. This questionnaire includes measures of attention to detail (10 items), attention switching (10 items), imagination (10 items), communication (10 items), and general social skills (10 items). For all items, participants indicated how well each statement described them on a scale from 1 (“definitely disagree”) to 4 (“definitely agree”). Negative worded items were reverse coded, so that higher scores for each item reflected more of each social engagement measure. Item responses within each measure were then combined to create five social engagement scores for each participant. Cronbach´s alphas in the current study for these social engagement measures ranged from 0.61 to 0.74.

#### Anxiety and depression

Symptoms of anxiety and depression were assessed using the anxiety (SLC-ANX) and depression (SLC-DEP) subscales of the Symptom Checklist (SLC-90)[36;37]. The SCL-ANX subscale is composed of the four items (i.e., “worrying too much”;”suddenly scared for no reason”; “nervousness or shakiness inside”; and “panic”), while the SCL-DEP is composed of six items (i.e., “feeling sad”; “feeling worthless”; “blaming yourself for things”; “feeling lonely”; “a feeling of being trapped”; and “suicidal thoughts”). Participants indicated how well each statement described them on a scale from 0 (“Not at all”) to 4 (“very much”). Item responses within the SCL-ANX were combined to create an overall anxiety-symptom score, and responses within the SLC-DEP were combined to create an overall depression-symptom score for each participant. Cronbach´s alphas for the SCL-ANX and SCL-DEP were 0.80 and 0.85, respectively.

#### Testosterone

Testosterone levels for all participants were measured by liquid chromatography tandem mass spectrometry, using Perkin Elmer´s CHS steroid MS kit. The lower limit of detection was 0.1nmol/L and the working range 0.2-100nmol/L.

### Analyses

In preliminary analyses, KS patients were first compared with controls in terms of testosterone, personality, social engagement, and anxiety and depression symptoms. Bivariate relationships were then calculated between the continuous study measures. In the primary analyses, two sets of three regressions—one set for anxiety, and one for depression—were conducted in which each of these two dependent measures (anxiety and depression) was regressed simultaneously on 1) personality traits, 2) social engagement measures, and 3) testosterone status, while controlling for KS status. Finally, a path analysis was conducted which included both anxiety and depression, as well as their respective bivariate predictors. Because path analysis is the functional equivalent of a series of multiple regressions in which each variable in the model is successively regressed on the other variables, it controls for multicollinearity and indicates the independent relationship between each of the factors in the model. As with the preceding regression analyses, variables in the path model were entered simultaneously—rather than hierarchically—and statistically significant relationships were included in the final path model, while non-significant associations were not.

Continuous data were tested for normality using Kolmogorov-Smirnov test, and those with distributions of Dα > 0.23 were considered non-normal, and were normalized using a loglinear (ln+1) transformation. Differences between groups were analyzed with parametric Student´s t-test for normally distributed data. Otherwise, data were analyzed using the Mann–Whitney–Wilcoxon test. Chi-Square tests were used for nominal variables. P-values lower than 0.05 were considered significant. SPSS version 19.0 (SPSS Inc., Chicago, Ill., USA) was used for statistical data analysis.

## Results

### Bivariate analyses

#### KS vs. controls

KS patients exhibited significantly higher neuroticism than controls, as well as significantly lower extraversion, openness to experience, and conscientiousness (Table 1). KS and controls did not differ in agreeableness. No differences were seen between KS patients treated with testosterone and untreated KS patients.

**Table 1:** KS patients vs. controls in terms of testosterone levels, personality, social engagement, anxiety and depression.

	KS	Controls		
*N*	69	69		
	Mean(SD)	Mean(SD)	p value	Cohen´s d
**Testosterone (nmol/L)**	17.4(11.2)	14.2(5.82)	0.04	0.36
**Personality**				
Neuroticism	^a^4.13(0.15)	^a^3.90(0.24)	<0.001	1.15
Extraversion	^a^3.73(0.31)	^a^3.95(0.29)	<0.001	0.73
Openness	^a^3.89(0.23)	^a^4.01(0.17)	0.001	0.60
Agreeableness	^a^3.91(0.21)	^a^3.87(0.23)	0.33	0.18
Conscientiousness	^a^3.81(0.25)	^a^3.91(0.25)	0.01	0.40
**Social Engagement**				
Attention to detail	^a^1.82(0.35)	^a^1.84(0.34)	0.75	0.06
Attention switching	^a^1.78(0.45)	^a^2.01(0.33)	0.001	0.58
Imagination	^a^1.82(0.39)	^a^2.04(0.28)	<0.001	0.65
Communication skills	^a^2.05(0.34)	^a^2.17(0.22)	0.01	0.42
Social skills	^a^1.95(0.41)	^a^2.14(0.31)	0.003	0.52
**Anxiety**				
Scl-anx	^a^1.29/1.39(0.73)	^a^0.90/1.10(0.62)	0.01	0.58
**Depression**				
Scl-dep	^a^1.30/1.39(0.93)	^a^0.85/0.69(0.83)	0.03	0.51

^a^Ln+1 transformed data

KS patients scored significantly lower than controls on attention switching, imagination, communication, and social skills. However, KS patients and controls did not differ in attention-to-detail, nor did KS patients treated with testosterone differ in social engagement from untreated KS patients.

KS patients reported significantly more anxiety and depression symptoms than controls, although there were no differences between KS patients treated with testosterone and those who were not.

KS patients had a significantly higher levels of testosterone than controls, reflecting the fact that the majority of these KS participants were receiving high doses of exogenous testosterone. Similarly, testosterone levels among KS patients receiving testosterone therapy were significantly higher than those not receiving this therapy.

#### Associations between non-KS variables

In addition to being related with each other, less neuroticism and more extraversion, conscientiousness, communication, attention switching and social skills were all associated with fewer anxiety and depression symptoms (Table 2). No significant differences were found between KS and controls in terms of either agreeableness or attention to detail (ps >.33). In addition, neither openness to experience nor imagination were associated with symptoms of anxiety or depression. Thus, these five variables were not included in subsequent multivariate analyses.

**Table 2:** Correlations between testosterone, personality traits, social engagement skills, anxiety and depression.

	Testosterone	Personality				Social engagement			
		Neuroticism	Extraversion	Openness	Conscientiousness	Attention switching	Imagination	Communication	Social skills
**Social Engagement**									
Attention switching	-0.239**	-0.519**	0.465**	0.246**	0.330**				
Imagination	0.083	-0.169*	0.321**	0.421**	0.067				
Communication	-0.145	-0.407**	0.481**	0.405**	0.336**				
Social skills	-0.125	-0.504**	0.714**	0.336**	0.316**				
** Overall score**	-0.219*	-0.494**	0.657**	0.400**	-0.381**				
**Depression**									
Scl-dep	0.127	0.640**	-0.339**	-0.071	-0.444**	-0.439**	-0.040	-0.298**	-0.323**
**Anxiety**									
Scl-anx	0.195*	0.554**	-0.283*	-0.022	-0.281**	-0.449**	-0.124	-0.259**	-0.257**

*p<0.05

**p<0.01.

### Multivariate analyses

#### Multiple regressions

When KS status and personality measures (i.e., neuroticism, extraversion, and conscientiousness) were combined to predict anxiety symptoms, the overall model was significant (R^2^ = .30, p<0.001), but neuroticism was the only significant individual predictor (Table 3). When anxiety was regressed on KS status and social engagement (i.e., social skills, communication and attention switching), the full model was again significant (R^2^ = .23, p < .001), with both KS status and attention switching emerging as significant predictors (ps<0.05). When KS status and testosterone levels were combined to predict anxiety, the full model was again significant (R^2^ = .09, p < .01), but this was attributable solely to the impact of KS status.

**Table 3:** Regression models predicting anxiety.

Independent variables	Standardized β coefficient	t-score	p-value
Personality			
KS Status	0.01	0.12	0.91
Neuroticism	0.52	4.93	<0.001
Extraversion	-0.04	-0.42	0.67
Conscientiousness	-0.01	-0.11	0.91
Overall model		F(4,137) = 14.35, p<0.001, R^2^ = 0.30	
Social Engagement			
KS Status	0.18	2.25	0.03
Social skills	0.05	0.50	0.62
Communication	-0.08	-0.81	0.42
Attention switching	-0.38	-4.00	<0.001
Overall model		F(4,136) = 9.64, p<0.001, R^2^ = 0.23	
Testosterone			
KS Status	0.30	3.33 0.001	
Testosterone status	0.03	0.32 0.75	
Overall model		F(2,137) = 6.26, p = 0.003., R^2^ = 0.09	

As shown in Table 4, KS status and personality measures (i.e., neuroticism, extraversion, and conscientiousness) combined to significantly predict depression symptoms (R^2^ = .36, p < .001), with neuroticism emerging as the lone significant predictor (p<0.001). Depression was also predicted by KS status and social engagement (i.e., social skills, communication, attention switching) (R^2^ = .22, p < .001), with attention switching as the significant predictor (p < .001). When combining KS status and testosterone status, the overall model was significant (R^2^ = .06, p = .014), but this was attributable to the impact of KS status (p = .01).

**Table 4:** Regression models predicting depression.

Independent variables	Standardized β coefficient	t-score	p-value
Personality			
KS Status	-0.08	-0.91	0.36
Neuroticism	0.55	5.41	<0.001
Extraversion	-0.08	-0.92	0.36
Conscientiousness	-0.05	-0.59	0.55
Overall model		F(4,136) = 18.12, p<0.001, R^2^ = 0.36	
Social Engagement			
KS Status	0.12	1.41	0.16
Social skills	-0.02	-0.22	0.83
Communication	-0.08	-0.84	0.40
Attention switching	-0.36	-3.83	<0.001
Overall model		F(4,135) = 9.29, p<0.001, R^2^ = 0.22	
Testosterone			
KS Status	0.24	2.56 0.01	
Testosterone status	-0.03	-0.31 0.76	
Overall model		F(2,136) = 4.44, p = 0.014, R^2^ = 0.06	

#### Path analysis

**Fig 1:**
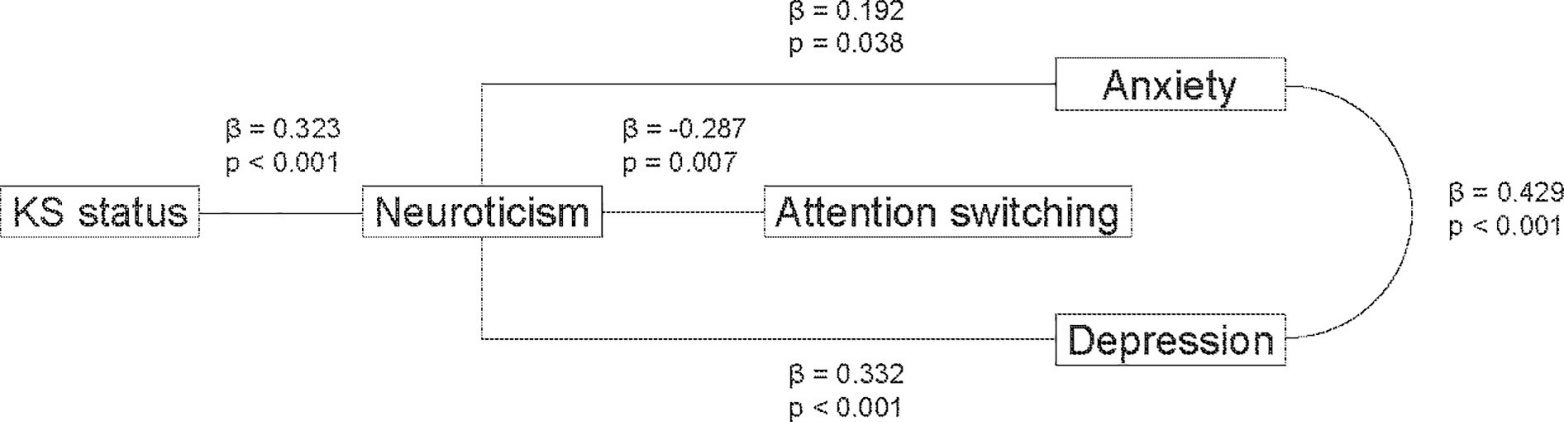
Path models of memory anxiety and depression with β standard coefficients and p-values.

When the significant personality and social-engagement predictors were combined with both anxiety and depression symptoms in an overall path analysis, neuroticism fully mediated the impact of KS status on both anxiety and depression (ps < .05), as seen in Fig 1. Neuroticism also mediated the effect of KS status on attention switching (ps < .01), which was not directly related to either anxiety or depression symptoms. The p-values of the non-significant associations ranged from .169 to .950.

## Discussion

This research examined the influence of personality, social engagement and testosterone treatment on anxiety and depression levels among KS patients. Although many of these measures were significant bivariate predictors of anxiety and depression symptoms, subsequent regressions indicated that only neuroticism and attention switching helped to explain the effect of KS on these indices of mental health. Finally, a combined path analysis found that KS increased both anxiety and depression symptoms through greater neuroticism.

These results are consistent with previous studies that have found more anxiety and depressive symptoms among patients with KS [3;4;7], but this is the first study to assess the possible underlying mechanism for these effects. Further, these findings indicate that, among the current study variables, neuroticism is the strongest (and only significant) mediating link between KS and both anxiety and depression. Neuroticism has also been found to be the strongest personality predictor of anxiety and depression in the general population [38–40], including the onset and developmental course of both anxiety-related attention deficit disorder and major depressive disorder [41–43]. Taken together, these results suggest that neuroticism has an impact across populations and levels of anxiety and depression.

In this research, higher levels of neuroticism also mediated KS patients’ deficits in attention switching. Attention switching is related to control processes of cognition such as perception, initiation, inhibition, and executive function[44;45]. As such, it is integral to the successful performance of activities involving multiple cognitive inputs, including decision-making, physical activity and social engagement, all of which have significant implications for personal, social and professional well-being. Although associated at the bivariate and regression analyses, attention switching was not related to anxiety or depression in the current path analysis. It could be that be that the earlier links between attention switching and anxiety and depression are attributable to other factors—most notably, neuroticism. Alternatively, it is possible that, with a larger sample size, these and other paths—both direct and indirect—may emerge.

The current results also have a number of practical implications for interventions to improve the well-being of individuals with KS. First, the central role of neuroticism suggests that it may be useful as a marker to identify KS patients at particularly high-risk for anxiety, depression and attention-switching problems, who would benefit most from assistance in these areas. A second possibility is suggested by studies finding that neuroticism levels can be raised or lowered through experience and/or training [46]. These results suggest that although neuroticism is developed early and is generally stable over time, it may nonetheless be mutable. If so, it may also be possible to improve mental health and other outcomes among KS patients (and perhaps the broader population) by reducing the neuroticism that influences them.

We did not find any influence of testosterone treatment on anxiety and depression levels among KS patients, which is in contrast with meta-analyses showing a positive impact of testosterone treatment on depressive symptoms in non-KS hypogonadal men from the general population [47–49]. The reason for this ´missing´ effect of testosterone treatment in KS seen in our study is not clear. Further prospective and longitudinal research is needed to address the influence of both hypogonadism and testosterone therapy on anxiety and depression among KS.

The limitations of this study include the cross-sectional nature of the research design. Although it takes advantage of the early development of personality—and even earlier onset of KS—it cannot determine the directionality or causal relationships between the study variables. Future longitudinal and experimental research will be useful to confirm the directionality of these effects, as well as their effectiveness in enhancing the well-being of KS patients, and perhaps other at-risk populations.

Another limitation of this study is that we did not assess socioeconomic variables. KS is associated with lower socioeconomic status—including lower educational and income, earlier retirement and fewer partnerships [50]—which in turn is related to higher levels of mental illness, including anxiety and depression [51;52]. Other socioeconomic-related factors that may also impact mental health—and help explain KS-related deficits—include low self-esteem, social support and psychological stress[53; 54]. Future research incorporating these factors among KS patients may further illuminate the mechanism for the increased prevalence of anxiety and depression among these patients.

As the first study to assess the influence of personality traits and social engagement on anxiety and depression among KS patients, this research suggests that personality, particularly neuroticism, plays a central role in attention switching and both measures of mental health. Future studies with larger sample sizes, longitudinal and experimental designs, and additional factors may help address many of the remaining questions, and further our understanding of KS-related deficits, and in turn, improve these and other important health outcomes.
